# New treatments for allergen immunotherapy

**DOI:** 10.1186/1939-4551-7-23

**Published:** 2014-09-24

**Authors:** Mübeccel Akdis

**Affiliations:** 1 Swiss Institute of Allergy and Asthma Research (SIAF) Davos, Obere Strasse 22, CH-7270 Davos Platz, Switzerland; 2 Christine Kühne – Center for Allergy Research and Education, Davos Platz, Switzerland

## Abstract

Allergen-specific immunotherapy (SIT) represents the only curative and specific way for the treatment of allergic diseases, which have reached a pandemic dimension in industrial countries affecting up to 20-30% of the population. Although applied for 100 years to cure allergy, SIT still faces several problems related to side effects and limited efficacy. Currently, allergen-SIT is performed with vaccines based on allergen extracts that can cause severe, often life threatening, anaphylactic reactions as well as new IgE sensitization to other allergens present in the extract. Low patient adherence and high costs due to long duration (3 to 5 years) of treatment have been commonly reported. Several strategies have been developed to tackle these issues and it became possible to produce recombinant allergen-SIT vaccines with reduced allergenic activity.

## 

Although performed for 102 years, allergen immunotherapy (AIT) faces several problems related to its efficacy, side effects, low patient adherence and the high costs due to long duration (3 to 5 years) of treatment [1,2]. The approaches to improve the efficacy and safety of vaccine-based AIT can be classified into different groups (Table 1).

**Table 1:** Novel vaccine development for allergen-SIT

**Type of the vaccine/approach**	**Description and mechanism**	**Current status**
**Bypassing IgE and targeting T cells**		
Fusion of major allergens [3] and chimeric allergens [4]	Major allergens or their fragments are fused and expressed as a single recombinant protein. IgE binding is attenuated, T cell reactivity is preserved. Preventive effect on generation of IgE is demonstrated in mice.	Effects in human cell cultures and mouse models.
Hypoallergenic hybrid molecules [5]	Derived from Der p 1 and Der p 2, reduced IgE reactivity of hybrid proteins, induce higher T cell proliferation responses.	Effects in human cell cultures and mouse models.
Fragments of major allergens [6]	Major allergen (Bet v 1) is divided into nonIgE binding fragments. IgE binding is attenuated and T cell reactivity is preserved.	A multi center clinical trial has been finalized.
Peptide immunotherapy [7-11]	NonIgE binding T cell epitope peptides (Fel d 1, Api m 1) have been used in cat and bee venom allergy.	Several promising clinical studies have been performed. Safe and tolerable in cat allergics
Unrefolded native or recombinant allergens [12]	Major recombinant allergens (Api m 1, Bet v 1) are not refolded to the native conformation. This decreases or abolishes IgE binding, but preserves T cell reactivity.	Several studies reported promising results.
Polymers of major allergens [6]	Major allergen (Bet v 1) is trimerized. Mast cell, basophil degranulation is attenuated, T cell reactivity is preserved in vitro.	A multi center clinical trial has been finalized.
**Reconstitution of the natural extract with multiple recombinant allergens**		
Mixture of several major recombinant allergens [13]	A mixture of five recombinant grass pollen allergens (Phl p 1, Phl p 2, Phl p 5a, Phl p 5b, Phl p 6) reduced symptoms and need for symptomatic medication in grass pollen allergic patients.	One clinical trial reported promising results.
**Allergens coupled to adjuvants**		
GpG oligonucleotide-conjugated allergens [14]	Major allergen (Amb a 1) is bound to a toll-like receptor 9-triggering CpG oligonucleotide.	A large multicenter clinical trial did not reach endpoints, but the clinical efficacy was robustly demonstrated.
Allergens coupled to virus-like particles [15]	Der p 1 coupled to highly repetitive virus capside-like recombinant particles	Rapid induction of high IgG antibody titers was observed in healthy human volunteers.
Carbohydrate-based particles [16]	Carbohydrate-based particles-bound rPhl p 5b induced a stronger antibody and cytokine responses.	In murine models.
Hypoallergenic vaccine based on allergen-derived peptides fused to hepatitis B PreS [17]	Recombinant fusion proteins show reduced allergenic activity in basophil activation and no IgE reactivity.	In murine models.
Monophosphoryl lipid A (MPL) formulated with allergoid [18]	Pollen allergoid formulated with the Th1-inducing adjuvant monophosphoryl lipid A (MPL) facilitates short-term SIT.	Results of clinical trials have been reported.
**Novel routes of administration**		
Intralymphatic vaccination [19]	Allergen-SIT vaccines administered directly into a lymph node. The aim is to deliver high amounts of allergens into secondary lymphatic organs.	Results of a clinical trial have been reported to show safety and efficacy.
Epicutaneous vaccination [20]	High numbers of antigen presenting cells (LCs), non-vascularized area, safe, needle-free, and potentially self-administrable.	Clinical trial in grass pollen-induced rhinoconjunctivitis demonstrated safety and efficacy.
**Fusion with immune response modifiers**		
Targeting FcγRII [21,22]	Fusion of allergens with human Fcγ has been reported to inhibit allergen-induced basophil and mast cell degranulation by crosslinking Fcγ and FcϵRI receptors.	Human cell cultures and mouse models.
Modular antigen translocation (MAT) vaccines [23]	The fusion of transactivator of transcription (Tat) peptide to both truncated invariant chain and allergens is able to target antigens to the nascent MHC II molecules in the trans-golgi compartment.	Clinical trial is finalized and demonstrated safety and efficacy with evidence of immune regulation.
**Combined treatment with immune response modifiers**		
Pre-treatment with anti-IgE mAb before SIT [24,25]	To reduce SIT induced side effects. To enable relatively rapid dose increase To use relatively high doses	Significantly fewer systemic allergic reactions, more patients were able to reach the target maintenance immunotherapy dose.

Targeting T cells to induce T cell tolerance and bypassing IgE binding to avoid IgE-mediated side effects is the first approach [26]. T cell tolerance appears to be one of the key mechanisms of action of AIT [27]. The conformation dependence of B cell epitopes and linearity of the amino acids sequence of T cell epitopes in the three dimentional structure of an allergen has been targeted in this group using allergen-fragments, fusions, hybrids and chimeras [3,4,6,26]. The prototype of this approach is peptide immunotherapy that utilizes linear T cell epitope peptides [7,8,28]. A major advantage of these T cell targeting approaches is that higher doses of allergens to be sequentially administered, which is required to induce T cell tolerance without the risk of anaphylaxis [12,26]. In addition, recently allergen-specific B regulatory (reg) cells have been demonstrated to be important. It appears that these Breg cells increase within the first 3 months of AIT and differentiate into IgG4-producing plasma cells. IL-10 coming from both B and T cells seem to be essential in IgG4 production [29].

The second approach is the use of recombinant allergens or their mixtures, with the aim of partially reconstituting an allergen extract. For example, a mixture of five recombinant grass pollen allergens showed effectiveness in reducing symptoms and the need for symptomatic medication in people with allergies to grass pollen [13]. All treated subjects developed strong allergen-specific IgG1 and IgG4 antibody responses. In another study of recombinant (r) Bet v 1 clinical improvement was observed accompanied by marked increases in Bet v 1-specific IgG levels, which were higher in the rBet v 1-treated group than in the birch and nBet v 1-treated groups. The recombinant allergen also avoided new sensitizations. New IgE specificities were induced in 3 of 29 patients treated with birch pollen extract, but in none of the 32 rBet v 1-treated or 29 nBet v 1-treated patients. No severe systemic adverse events were observed in the rBet v 1-treated group [30]. There have been more than 10 clinical trials-all showing increased clinical efficacy compared to placebo group-completed by using recombinant allergens during the last decade [31,32]. Recombinant allergens provide a significant advantage in the problems of standardization of allergen extracts.

Another important approach is to physically couple allergens to stimulators of the innate immune response. This area is very open to future developments, as there are infinite possibilities for combinations owing to the existence of multiple immune stimulators and methods for coupling [14-18]. Notably, the time, intensity and tissue location of stimulation of the innate immune response by the allergen is decisive in the induction of tolerance or immune activation. For example, house dust mites can use Toll-like receptor 4 triggering in airway structural cells to induce asthma/like inflammation in mice [33]. It is expected that novel adjuvants may induce allergen-specific T cell tolerance with relatively less allergen doses and may direct the unwanted Th2 response towards a Tregulatory response in an earlier time frame. However, so far these approaches did not end up with a fruitful outcome. The generation and maintenance of allergen-specific T-cell tolerance is a key step in healthy immune responses to allergens and successful AIT. Breaking of peripheral T-cell tolerance to allergens can lead to the development of allergies, but the mechanisms are not completely understood. It was recently demonstrated that certain innate immune response signals and proinflammatory cytokines, such as IL-1β, IL-6, TLR-4-ligands and TLR8-ligands break allergen-specific CD4 T-cell tolerance in normally unresponsive subjects, which might lead to the development or exacerbation of allergic diseases after encountering microbes or inflammatory conditions. The usage of novel adjuvants should be considered also in this perspective [34].

The fourth approach is varying routes of vaccine administration. A meta-analysis of the double-blind, placebo-controlled trials has shown that sublingual immunotherapy (SLIT) is clinically efficacious with a treatment benefit approximately half of that achieved with subcutaneous AIT [35]. Sustained disease-modifying effects of this type of therapy have been confirmed in large-scale randomized, double-blind, placebo-controlled trials as well as in children [36,37]. The immunological mechanisms of SLIT seem to be similar to subcutaneous AIT, although the magnitude of the change in most parameters is modest or no changes have been observed. Immune tolerance induced by SLIT is associated with Treg cells, IL-10 production, increases in sublingual Foxp3-expressing cells and elevated allergen-specific IgG4, IgA and serum inhibitory activity for IgE-facilitated allergen binding to B cells [38,39]. Recently, allergen-specific FOXP3+ Treg cells have been found in human lingual and palatine tonsils in humans, and these cells may participate in oral tolerance and SLIT [40]. Oral mucosal tissue is naturally tolerogenic, and does not show acute inflammation despite high levels of bacterial colonization, and shows rapid wound healing in the absence of scarring. A C-type lectin receptor, SIGNR-1 (also called Cd209b), helps to condition dendritic cells in the gastrointestinal lamina propria for the induction of oral tolerance in a model of food-induced anaphylaxis. These results suggest that sugar-modified antigens might be used to induce oral tolerance by targeting SIGNR1 and lamina propria DCs [41]. Recently the intralymphnode and epicutaneous routes have been tested. Both routes showed similar efficacy to subcutaneous injection immunotherapy in grass pollen allergy, but less applications and lower total doses of allergen were required using these routes [19,20]. Intralymphatic administration induced T cell responses with strong cytotoxic activity and IFN-γ production that conferred long-term protection against viral infections and tumors.

In another approach, the coaggregation of FcϵRI and FcγRII receptors that inhibit FcϵRI signaling and the fusion of allergens to human Fcγ have been reported to inhibit allergen-induced basophil and mast cell degranulation by crosslinking Fcγ and FcϵRI receptors [21,22]. In a different approach, the major cat allergen Fel d 1 was cloned and expressed together with a human immune deficiency virus protein, TAT-derived membrane translocation domain, and a truncated peptide of the invariant chain (modular antigen translocation (MAT)-Fel d 1) [42]. This MAT-Fel d 1 vaccine is efficiently internalized and potently presented to T cells by antigen-presenting cells, and induced T cell responses at doses which were approximately 100x lower than those of the native allergens. In a double-blind, placebo-controlled clinical trial, the MAT-Fel d 1 vaccine with alum adjuvant was administered in three increasing doses (1 μg, 3 μg, 10 μg) into inguinal, lymph nodes at 4 week intervals. In addition to a good safety profile, individuals who were allergic to cats and treated with the MAT-Fel d 1 vaccines became clinically tolerant to nasal challenge of cat dander extract in parallel with increased serum IgG4 [23]. The findings were not reproduced in another study of intralymphatic immunotherapy [43].

In addition to physical fusion, there is also a huge potential for combining o conventional and novel methods of AIT with immune response modifiers. For example, anti-IgE combined with AIT has been evaluated in several studies [24], and resulted in a significant decrease in the risk of anaphylaxis caused by rush immunotherapy (a rapid dose increment approach to reach the maintenance dose as quick as possible) and improved rescue medication scores (the need for a rescue medication to suppress the symptoms: for example anti-histamines for allergic rhinitis) of AIT with a good safety profile [24,44]. In a recent study, eighteen weeks' treatment of omalizumab in combination with AIT in patients with seasonal allergic rhinitis and comorbid seasonal allergic asthma reduced the symptom load during the treatment period but showed no prolonged effect during treatment with AIT only [25]. Combination strategies with biological immune response modifiers are expected to substantially expand the treatment scope of AIT. Overall, there has been tremendous progress in addressing the challenge of improving efficacy and safety of AIT, which is still the only approach for curing allergic diseases. In addition, this strategy is still open to further developments, which are expected to improve its application to other diseases related to dysregulation of the immune system.

## Conclusion

Currently, allergen-specific immunotherapy is the only available curative treatment of allergic diseases as it can induce long-term allergen-specific immune tolerance using multiple mechanisms. AIT is beneficial for a significant fraction of the treated individuals, however life-threatening side effects can occur. Due to the long duration of the treatment there is a problem with full adherence to the therapy. Whereas it can be curative in some individuals, there are non responders to treatment. To overcome these problems, it is crucial to develop advanced vaccines. In addition, the discovery of reliable biomarkers to select patients with a good clinical response is important. Notably, the AIT-based curative approaches may also be promising for the prevention of allergic disease. A greater understanding of the underlying disease mechanisms in allergic diseases could bring the possibility of cure in a larger patient population nearer to reality.

## Competing interests

The author declares she has no competing interests.
